# A gas sensor array for the simultaneous detection of multiple VOCs

**DOI:** 10.1038/s41598-017-02150-z

**Published:** 2017-05-16

**Authors:** Yumin Zhang, Jianhong Zhao, Tengfei Du, Zhongqi Zhu, Jin Zhang, Qingju Liu

**Affiliations:** School of Materials Science and Engineering, Yunnan Key Laboratory for Micro/nano Materials & Technology, Yunnan University, Kunming, 650091 China

## Abstract

Air quality around the globe is declining and public health is seriously threatened by indoor air pollution. Typically, indoor air pollutants are composed of a series of volatile organic compounds (VOCs) that are generally harmful to the human body, especially VOCs with low molecular weights (less than 100 Da). Moreover, in some situations, more than one type of VOC is present; thus, a device that can detect one or more VOCs simultaneously would be most beneficial. Here, we synthesized a sensor array with 4 units to detect 4 VOCs: acetone (unit 1), benzene (unit 2), methanol (unit 3) and formaldehyde (unit 4) simultaneously. All units were simultaneously exposed to 2.5 ppm of all four VOCs. The sensitivity of unit 1 was 14.67 for acetone and less than 2.54 for the other VOCs. The sensitivities of units 2, 3 and 4 to benzene, methanol and formaldehyde were 2 18.64, 20.98 and 17.26, respectively, and less than 4.01 for the other VOCs. These results indicated that the sensor array exhibited good selectivity and could be used for the real-time monitoring of indoor air quality. Thus, this device will be useful in situations requiring the simultaneous detection of multiple VOCs.

## Introduction

It is an indisputable fact that volatile organic compounds (VOCs), which are common air pollutants, are harmful to the human body^1–3^. Indoor environmental pollution caused by VOCs has become an important issue. For example, acetone, which is a widely used solvent in industry and laboratories, can volatilize easily and affect human health when its concentration exceeds 173 ppm^4^. Additionally, benzene and its homologs, such as toluene and xylene, are known to be toxic to the hematopoietic system (hematotoxicity) and to cause leukemia^5^. Methanol possesses strong toxicity, especially to the blood and nervous system. Furthermore, previous studies have shown that methanol is potentially harmful to human optic nerves and retinas^6, 7^. Formaldehyde has been classified as a mutagen and possible human carcinogen by both the US Environmental Protection Agency and the World Health Organization because of its toxicity, anaphylactic potential and harmful accumulation in the environment^8, 9^. Effort has been expended to detect these harmful VOCs, and some of the relevant works are summarized in Table S1 (Supporting Information). Currently, detecting a particular VOC requires a specific gas sensor, and no device made with same material or substrate able to simultaneously detect multiple VOCs has been reported. In this paper, we report a gas sensor array containing 4 units that respectively respond to acetone, benzene, methanol and formaldehyde. Therefore, this array can simultaneously detect these four types of VOCs. The 4 units are all based on Ag-doped LaFeO_3_ (ALFO) and can thus be fabricated on the same substrate, which greatly contributes to the significance and convenience of the application of the device.

In contrast to some single-metal oxide semiconductors, LaFeO_3_ (LFO) is a common perovskite-type oxide that exhibits semiconducting behavior^10^ and is a promising material with an abundance of functionalities, especially in the field of gas sensing. LFO possesses great potential for detecting pollutant gases because of its specific chemical and physical characteristics, including its large surface area, rich active oxygen lattice, good thermostability^11^, controllable structure^12^, and strong reducibility^13–17^. Thus, LFO is a more attractive gas-sensing material than other metal oxides. In addition, when LFO is doped with Ag (ALFO), some Ag is present that can act as a catalyst in the matrix. Indeed, some of the Ag fills areas between the grains of the matrix and works to decrease the contact potential barrier and enhance the interfacial effects, leading to a lower resistance, and thus, a lower operating temperature^18^. Therefore, ALFO was selected as the material for the units developed in this study.

Most importantly, the selectivity of ALFO toward acetone, benzene, methanol and formaldehyde can be modulated via the molecular imprinting technique (MIT). The MIT results in a predesigned molecular recognition capability that can be used to build robust sensors^19–22^. In this approach, the shape and functionality of a template can be transcribed onto microporous materials. The configuration of the functional groups in the template can be memorized within the host polymers. Today, this field is dominated by the recognition and separation of organic macromolecules, such as proteins^19–21^ (molecular weight: 40–220 kDa) and enzymes^22^ (130–140 kDa). However, for small organic molecules, such as VOCs (molecular weight <100 Da), no relevant research has yet been reported.

**Figure 1:**
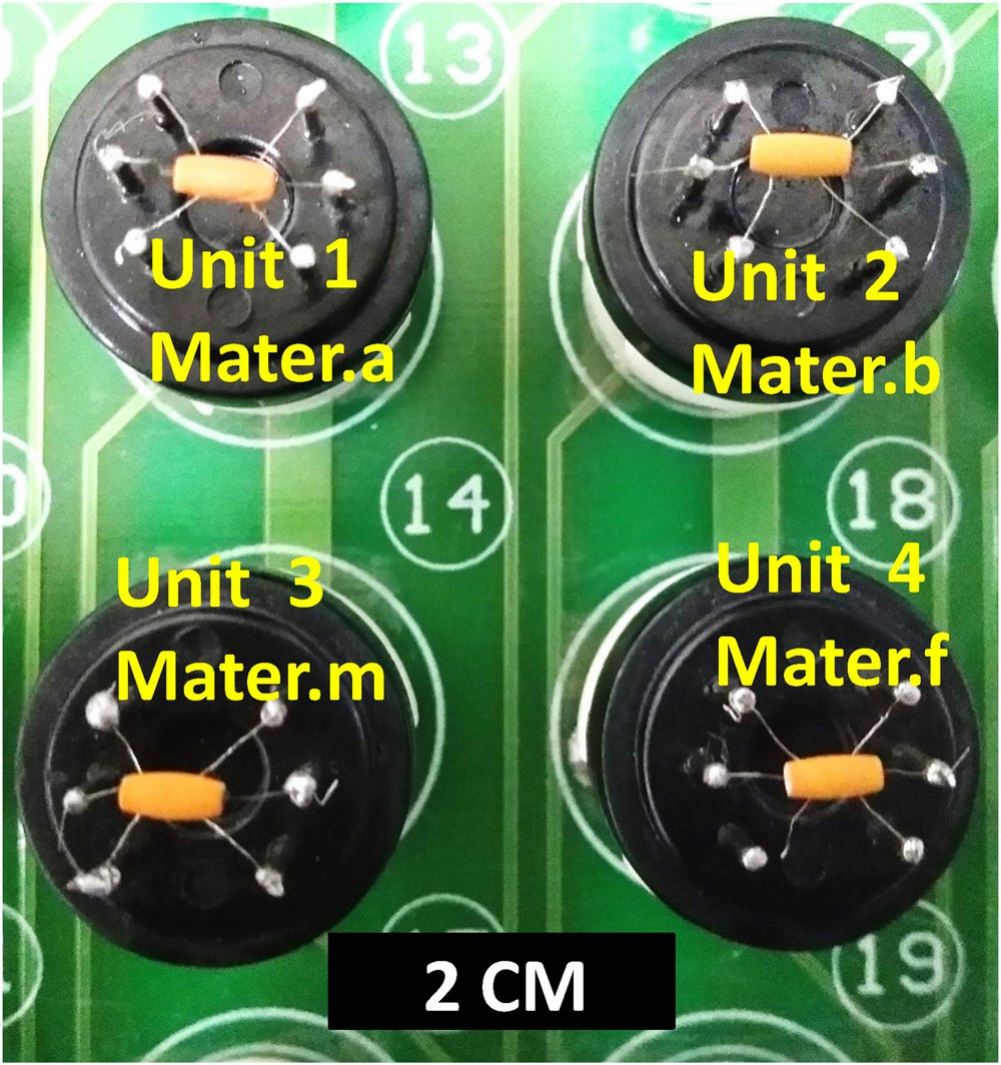
Photograph of the sensor array. Each unit in the array is a heater-type sensor. The black scale bar represents 2 cm.

Based on the MIT, using acetone as a template and choosing an appropriate acetone-related functional monomer, ALFO was prepared as an acetone gas-sensing material (hereafter abbreviated as Mater.a). Similarly, using benzene, methanol and formaldehyde as templates and choosing appropriate functional monomers, ALFO can be prepared as benzene (Mater.b), methanol (Mater.m) and formaldehyde (Mater.f) gas-sensing material. Subsequently, the four materials were brush-coated onto a sensor array, as shown in Fig. 1. Each unit of the array can be described as follows: Unit 1 was fabricated with Mater.a for acetone detection, unit 2 was fabricated with Mater.b for benzene detection, unit 3 was fabricated with Mater.m for methanol detection and unit 4 was fabricated with Mater.f for formaldehyde detection. As a result, the array is capable of monitoring acetone, benzene, methanol and formaldehyde or any combination of these gases simultaneously. Based on these results, using any gas molecule as a template, ALFO can be prepared as a sensor to detect the template molecule.

The structures of Mater.a, Mater.b, Mater.m and Mater.f were orthogonal perovskite (for the X-ray diffraction [XRD] patterns, see Supporting Information Figure S1). These structures included only one phase of LFO because the amount of the dopant (Ag) was so small (mol (Ag):mol (LFO) = 1%) that it could not be detected. Additionally, the template, functional monomer and initiator were removed after sintering^23^, as a result, template, functional monomer and initiator could not be detected either. The microtopographies of Mater.a, Mater.b, Mater.m and Mater.f were spherical and uniform in size, with particle sizes in the range of 20–70 nm (for the transmission electron microscopy [TEM] images, see Supporting Information Figure S2). The small particles were ideal because of their specific surface area (SSA, see Supporting Information Table S2). Materials with larger SSAs can adsorb analytes easily and therefore enhance the sensitivity of the resulting device.

To investigate the interactions of the template, functional monomer and crosslinker, Fourier transform infrared (FT-IR) spectra of ALFO, acetone, benzene, methanol and formaldehyde and the related functional monomers N,N’-methylenebisacrylamide (MBA), formaldehyde (HCHO), methacrylic acid (MAA) and acrylamide (AM) were obtained (see Supporting Information Figure S3).

**Figure 2:**
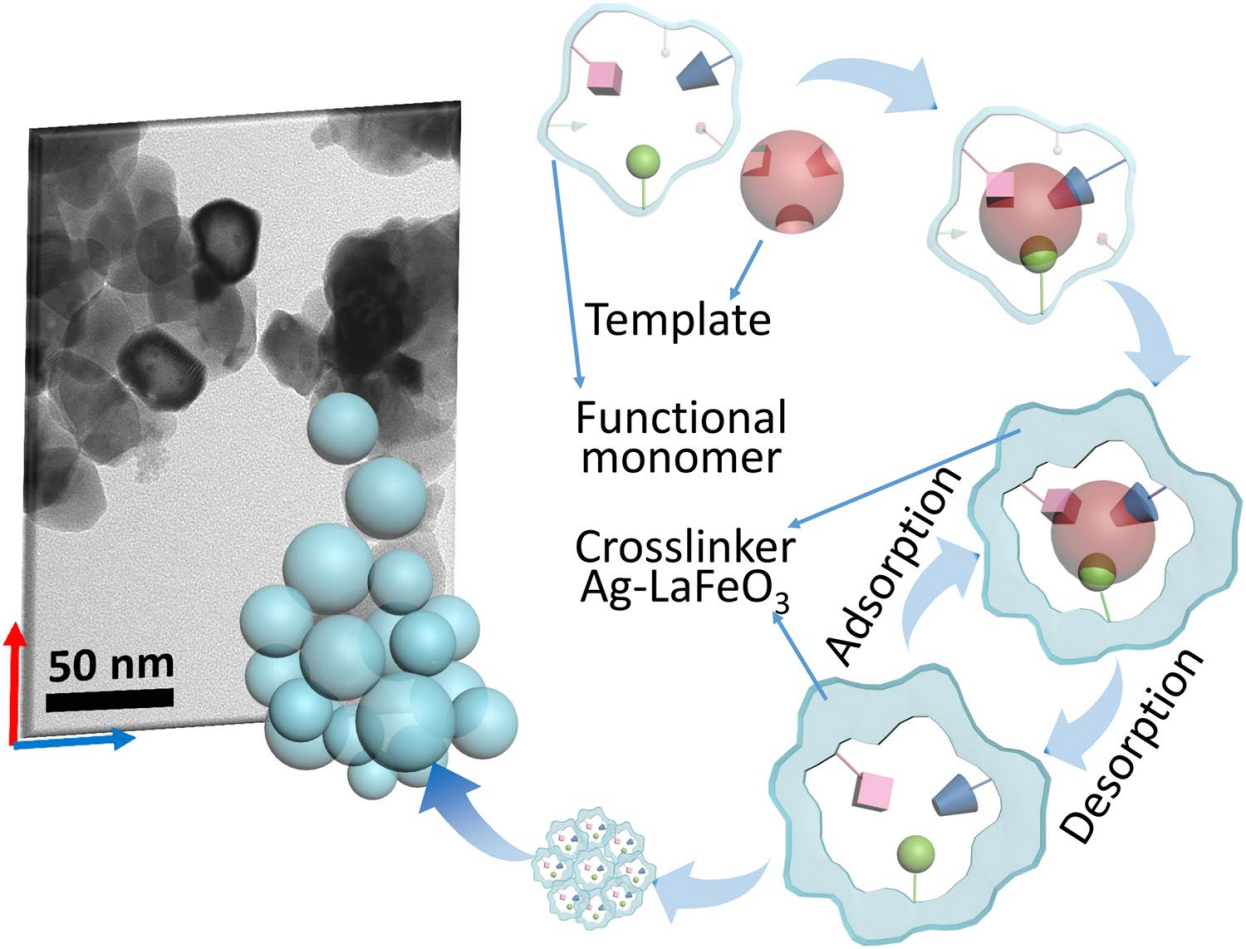
Schematic of the molecular imprinting process.

LFO is a typical p-type semiconductor in air, and its gas-sensing mechanism is based on the changes of the resistance before and after exposure to a test gas^24, 25^. The gas sensitivity was defined as the ratio of the electrical resistance in the gas (Rg) to that in air (Ra). After the ALFO was polymerized with MBA, the gas-sensing mechanism was similar to that of LFO because LFO was the only phase in Mater.a (see Supporting Information Figure S1 for the XRD patterns). The mechanism underlying the specific recognition of acetone is illustrated in Fig. 2. A number of recognition cavities complementary to acetone in their shape, size and chemical functionality were able to selectively adsorb acetone; thus, the sensor selectivity for acetone could be modulated. As illustrated in Fig. 2, when the template (acetone) was mixed with the functional monomers (MBA), acetone interacted with MBA via hydrogen bonds, and then an acetone-MBA complex was formed^17^. The resulting complex was subsequently copolymerized with a large excess of crosslinker. Finally, after removing the template, recognition cavities complementary to the acetone molecules were formed on the ALFO. These cavities were accessible sites for acetone, and they possessed high recognition and binding abilities for acetone only, ultimately resulting in good selectivity for acetone. Therefore, the selectivity of ALFO could be modulated toward acetone. Compared to ALFO, Mater.a exhibited better selectivity for acetone while maintaining high sensitivity. Subsequently, benzene, methanol and formaldehyde were each used as template molecules, and the selectivity modulation of Mater.b, Mater.m and Mater.f was performed as for Mater.a.

Selectivity is an important property of gas sensors and is the ability of a sensor to respond to a particular gas in the presence of other gases. We measured the sensitivity of the array to eight types of volatile analytes: acetone, formaldehyde, methanol, toluene, gasoline, ammonia, benzene and ethanol. During these tests, the operating temperature and relative humidity were 125 °C and 45%, respectively (Supporting Information Figures S4 and S5). Figure S4 shows that the ideal relative humidity was between 20% and 50%. The relative humidity was chosen based on the range in which humans can live comfortably (i.e., conditions in which a sensor for monitoring the air quality can be required). Therefore, the upper and lower limits could not be too high or too low, respectively. In this range, the array performed well and was stable at values of 20–50%. However, when the relative humidity was increased to 70%, and then further to 90%, water molecules occupied most of the testing space and surfaces of the sensing materials, passivating the sensing materials and blocking the VOCs from reaching the material surface. As a result, the sensitivity of the sensing materials to the same concentration of VOCs (2.5 ppm) decreased. Therefore, 45% humidity was chosen to for the tests. Regarding the operating temperature, for a reaction between a target gas and the adsorbed oxygen, a certain activation energy is required, which can be provided by increasing the reaction temperature. Hence, a high response can only be obtained at a suitable temperature using appropriate sensing materials and target gases. For ALFO, at room temperature, the adsorbed VOC molecules were not sufficiently activated to overcome the activation energy barrier and thus react with the adsorption oxygen species. By contrast, at high temperatures, the gas adsorption was too difficult and could not be compensated for by the increased surface reactivity. Thus, we chose 125 °C as the optimum operating temperature for all subsequent experiments. In addition, at 2.5 ppm, the analytes showed perfect stability and repeatability. Therefore, the analyte concentration was 2.5 ppm for all tests.

**Figure 3:**
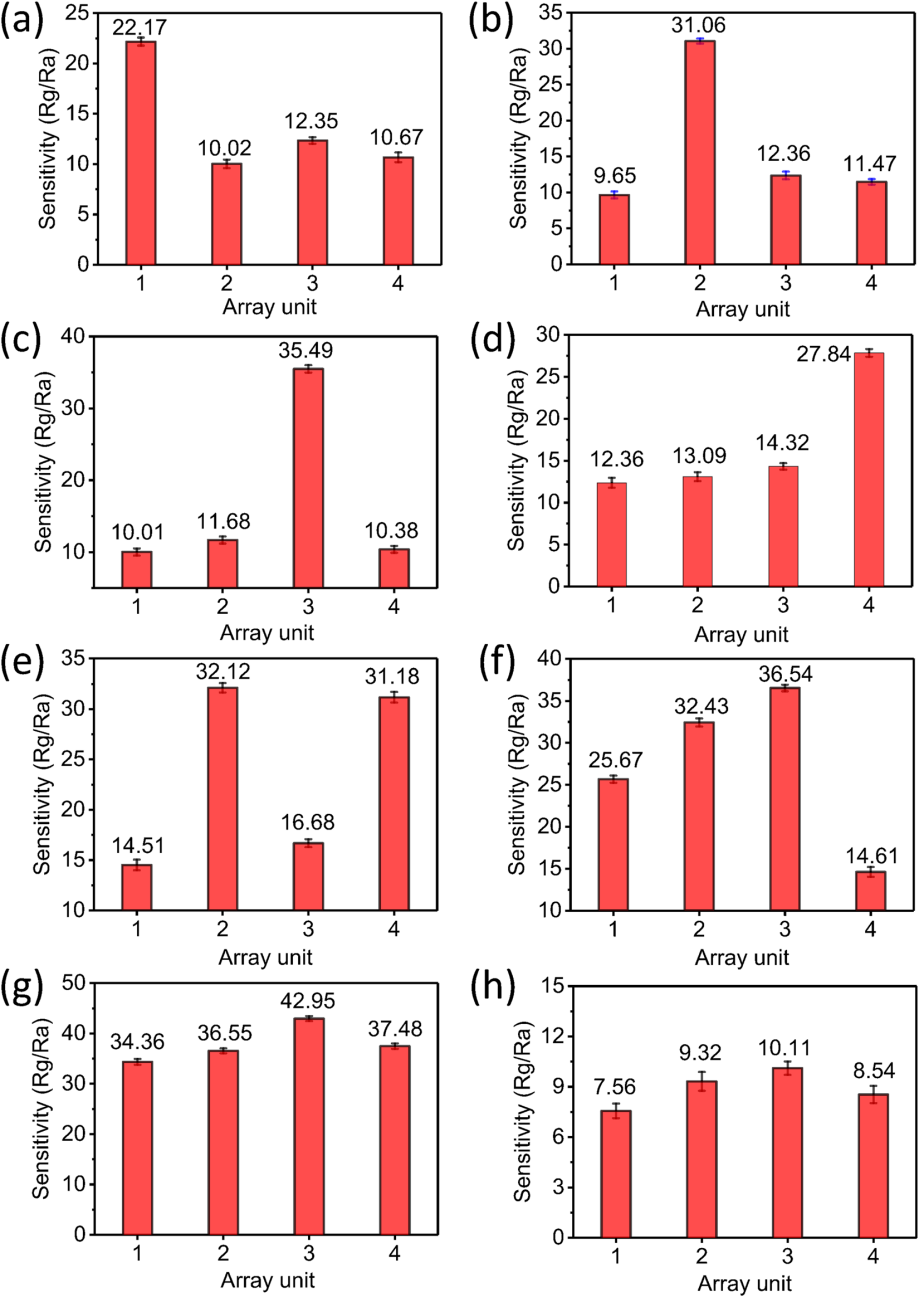
The sensitivity of the array to the target analytes and interferents (ethanol, gasoline, toluene and ammonia). (**a**) Acetone + interferents: unit 1 responded markedly; (**b**) benzene + interferents: unit 2 responded markedly; (**c**) methanol + interferents: unit 3 responded markedly; (**d**) formaldehyde + interferents: unit 4 responded markedly; (**e**) benzene + formaldehyde + interferents: units 2 and 4 responded simultaneously; (**f**) acetone + benzene + methanol + interferents: units 1, 2 and 3 responded simultaneously; (**g**) all eight analytes: all of the units responded simultaneously; and (**h**) interferents: no notable response was observed.

Acetone, benzene, methanol and formaldehyde were the target analytes, and ethanol, toluene, ammonia and gasoline were the interferents. Ethanol, toluene, gasoline and ammonia were chosen as interferents because the sensitivities of most oxide semiconductors to these analytes are known to be high^26–29^. Additionally, these analytes are ubiquitous gases^30–33^. The interferents mentioned hereafter in the text or in the figure captions refer to a mixture of 2.5 ppm ethanol + 2.5 ppm toluene + 2.5 ppm gasoline + 2.5 ppm ammonia. To assess the ability of the array to function as a gas sensor in a real situation, it was tested in air. The sensitivity of the array to each analyte individually (Supporting Information Figure S6) and to mixtures of the target analytes (acetone, benzene, ethanol, formaldehyde or any combination thereof) and the interferents (Fig. 3) was tested systematically. The composition of the test analytes and the sensitivity of each unit to these test analytes are also shown in Table 1. When the test mixture contained only acetone and the interferents, unit 1 (see Fig. 1) exhibited a higher response (Fig. 3a). The sensitivity of unit 1 was 22.17, whereas those of units 2, 3 and 4 were 10.02, 12.35 and 10.67, respectively. Likewise, when the test mixture contained only benzene and the interferents, unit 2 showed a strong response (Fig. 3b), with a sensitivity of 31.06. This value was approximately 20 higher than those of units 1, 3 and 4. For methanol (Fig. 3c), the sensitivity of unit 3 was 35.49, twice higher than those of units 1, 2 and 4. Finally, for formaldehyde (Fig. 3d), the sensitivity of unit 4 was 27.84, which was twice those of units 1, 2 and 3. Namely, units 1, 2, 3 and 4 exhibited good selectivity for acetone, benzene, methanol and formaldehyde, respectively. When the array was exposed to a mixture of the 8 analytes (Fig. 3g), the sensitivity of each unit was approximately 30, which is much higher than the values observed when the array was exposed to only the interferents (Fig. 3h). In summary, when the test gas contained acetone, benzene, ethanol, formaldehyde or any combination thereof, the corresponding unit(s) in the array exhibited marked responses. Hence, this array achieved the simultaneous detection of multiple VOCs; that is, acetone, benzene, ethanol and formaldehyde. Furthermore, the number of units in such as sensor array could be added or removed as desired. In general, for use in a house, a sensor array containing two units-one for formaldehyde detection and one for benzene detection—would be sufficient (Fig. 3e). By contrast, for laboratory use, units for detecting acetone, methanol, benzene and other VOCs will be needed (Fig. 3f).

**Table 1:** The compositions of the test analytes and the sensitivity of the 4 units in each test.

Composition of the test analyte	Sensitivity of each unit in the array			
	Unit 1	Unit 2	Unit 3	Unit 4
benzene only	2.15	**18.64**	2.10	2.27
ethanol only	2.34	3.67	4.31	2.69
methanol only	2.46	3.15	**20.98**	2.54
gasoline only	2.54	3.78	2.57	1.24
toluene only	1.25	3.85	3.26	2.55
acetone only	14.**67**	1.04	2.85	1.96
ammonia only	1.36	1.96	2.74	4.01
formaldehyde only	2.61	4.01	2. 43	**17.26**
acetone + interferents	**22.17**	10.02	12.35	10.67
benzene + interferents	9.65	**31.06**	12.36	11.47
methanol + interferents	10.01	11.68	**35.49**	10.38
formaldehyde + interferents	12.36	13.09	14.32	**27.84**
benzene + formaldehyde + interferents	14.51	**32.12**	16.86	**31.18**
acetone + benzene + methanol + interferents	**25.67**	**32.43**	**36.54**	14.61
all 8 types of analyte	**34.36**	**36.55**	**42.95**	**37.48**
interferents	7.56	9.32	10.11	8.54

**Figure 4:**
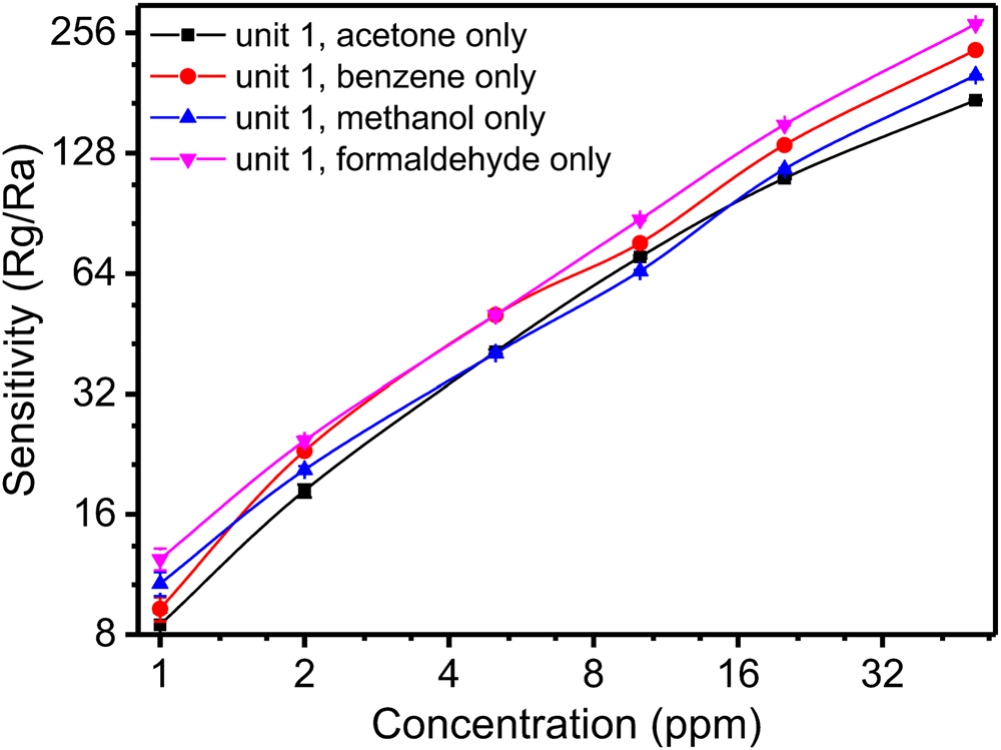
The relationship between sensitivity of each unit and the concentration of each target analyte.

Then, the relationship between the sensitivity and concentration was investigated (Fig. 4). The temperature and relative humidity during the tests were 125 °C and 45%, respectively. The sensitivities of the four units increased linearly as the concentration of each target analyte increased. This phenomenon occurred because the surface of each unit contained many analyte-adsorbing vacancies. As the concentration of the test analyte increased, the quantity of the adsorbed analyte on the surface of the unit increased, and the electrons produced by that unit also increased. Thus, for the p-type semiconductors, the resistance values of the units increased, eventually increasing the sensitivity. For gas concentrations from 1 ppm to 50 ppm, the sensitivity of the units increased linearly, indicating that such array can be used for the continuous real-time monitoring of VOCs at low concentrations. The response time and recovery time of unit 1 to 2.5-ppm acetone were 10 s and 100 s, respectively. The response time and recovery time for unit 2 (2.5-ppm benzene), unit 3 (2.5-ppm methanol), and unit 4 (2.5-pm formaldehyde) were 15 s and 100 s, 15 s and 95 s, and 10 s and 95 s, respectively (Supporting Information, Figure S7).

The MIT enabled the selectivity of ALFO to be modulated to detect acetone, benzene, methanol and formaldehyde. Specifically, acetone, benzene, methanol and formaldehyde could be selectively recognized based on a single type of gas-sensing material, and thus, a low limit of detection, high sensitivity and high selectivity could be achieved simultaneously. Thus, the MIT can modulate the selectivity of certain metal oxides, facilitating the fabrication of larger arrays containing more units to detect more VOCs at the same time. These findings present a new feasible method for applying ALFO to many other varieties of gas sensors via the MIT.

## Methods

Preparation of the crosslinker: All the chemicals were analytical-grade reagents and were used as received from Tianjin Kermel Chemical Reagents Development Center. To prepare the crosslinker, 9.9 mmol of La(NO_3_)_3_ **·**6H_2_O, 10.0 mmol of Fe(NO_3_)_3_ **·**9H_2_O and 10.0 mmol of citrate were dissolved in 100 mL of distilled water to form solution A. AgNO_3_ (0.1 mmol) was dissolved in 10 mL of distilled water and added dropwise (12 drops/min) to solution A. Subsequently, polyethylene glycol was added. The final mixed solution was stirred at 80 °C for 8 h and then placed in a microwave chemical device (CEM, USA) at 75 °C for 2 h. Thus, the ALFO sol was formed and used as a crosslinker in the molecular imprinting process.

Preparation of the ALFO-based Mater.a, Mater.b, Mater.m, and Mater.f:

1. Preparation of Mater.a: Acetone was used as the template, MBA was used as the functional monomer, azodiisobutyronitrile (AIBN) was used as the initiator and ALFO sol was used as the crosslinker. A schematic diagram of the hydrogen bonding between acetone and MBA is shown in Fig. 5a. The molar ratio of the functional monomer and crosslinker was defined as x = mol (MBA):mol (ALFO sol) = 5:100. Then, 1.0 mmol of acetone was mixed with 0.5 mmol of MBA, treated with ultrasonication for 30 min, and then left for 8 h to form solution B_a_. Subsequently, 1.0 mmol of AIBN was dissolved in 20 mL of acetone and mixed with solution B_a_ and ALFO sol (10.0 mmol). The final mixture was treated with ultrasonication for 30 min, stirred at 50 °C for 12 h under a nitrogen atmosphere under reflux and then dried. Finally, the xerogel was heated at 800 °C for 2 h, yielding Mater.a.

**Figure 5:**
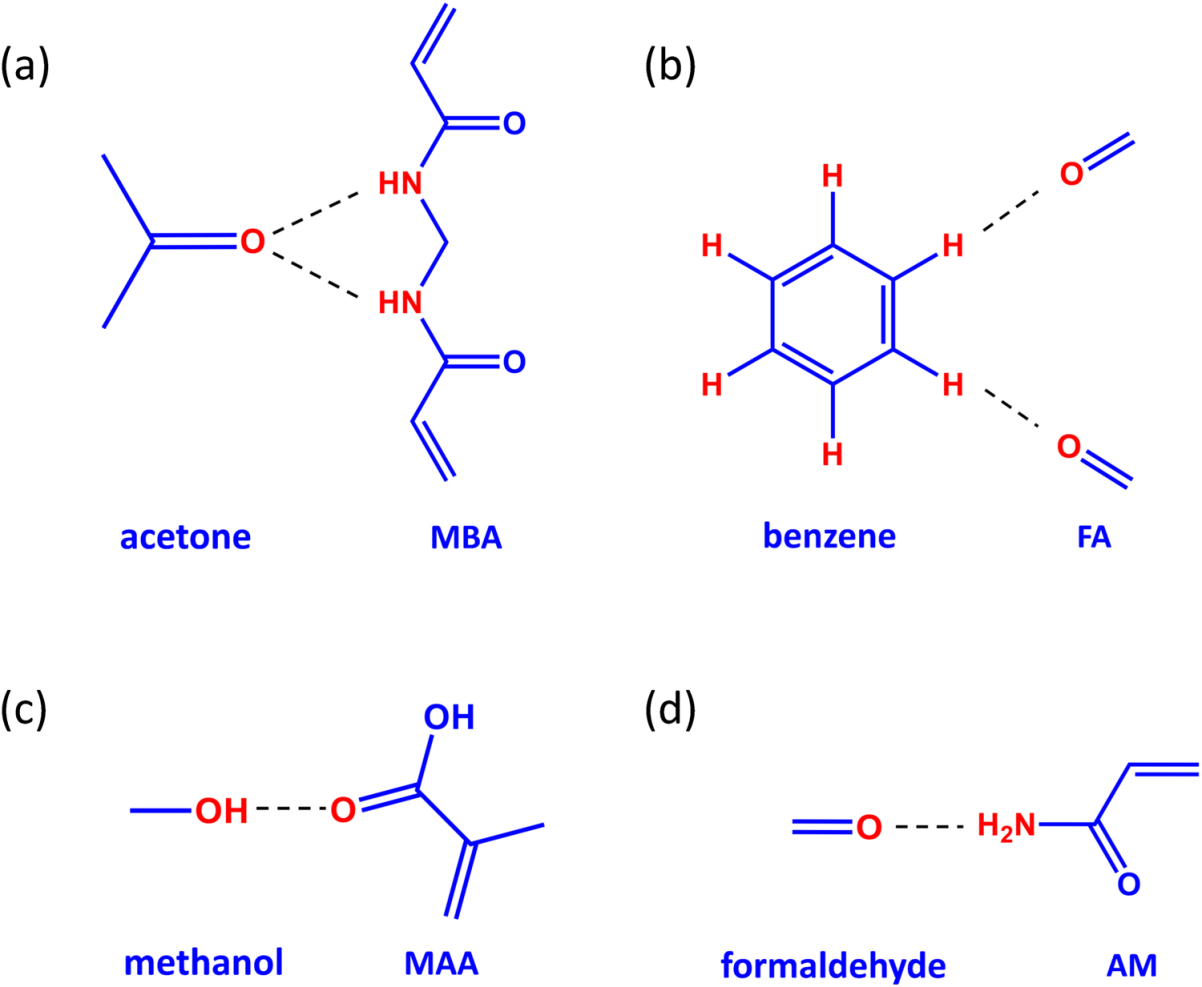
The combinations of the template and functional monomer: (**a**) Acetone and MAA for Mater.a (**b**) Benzene and formaldehyde (FA) for Mater.b (**c**) Methanol and MAA for Mater.m (**d**) Formaldehyde and AM for Mater.f.
2. Preparation of Mater.b: Benzene was used as the template, FA was used as the functional monomer, AIBN was used as the initiator and ALFO sol was used as the crosslinker. A schematic diagram of the hydrogen bonding between benzene and FA is shown in Fig. 5b. The molar ratio of the functional monomer and crosslinker was defined as y = mol (HCHO):mol (ALFO sol) = 60:100. First, 1.0 mmol of benzene was mixed with 6.0 mmol of FA, treated with ultrasonication for 30 min, and left for 8 h to form solution B_b_. Then, 1.0 mmol of AIBN was dissolved in 20 mL of benzene and mixed with solution B_b_ and ALFO sol (10.0 mmol). The final mixture was treated by ultrasonication for 30 min, stirred at 50 °C for 12 h under a nitrogen atmosphere under reflux and then dried. Finally, the xerogel was heated at 800 °C for 2 h, yielding Mater.b.
3. Preparation of Mater.m: Methanol was used as the template, MAA was used as the functional monomer, AIBN was used as the initiator and ALFO sol was used as the crosslinker. A schematic diagram of the hydrogen bonding between methanol and MAA is shown in Fig. 5c. The molar ratio of the functional monomer and crosslinker was defined as z = mol (MAA):mol (ALFO sol) = 40:100. First, 1.0 mmol of methanol was mixed with 4.0 mmol of MAA, treated with ultrasonication for 30 min, and left for 8 h to form solution B_m_. Then, 1.0 mmol of AIBN was dissolved in 20 mL of methanol and mixed with solution B_m_ and ALFO sol (10.0 mmol). The final mixture was treated with ultrasonication for 30 min, stirred at 50 °C for 12 h under a nitrogen atmosphere under reflux and then dried. Finally, the xerogel was heated at 800 °C for 2 h, yielding Mater.m.
4. Preparation of Mater.f: Formaldehyde was used as the template, AM was used as the functional monomer, AIBN was used as the initiator and solution B was used as the crosslinker. A schematic diagram of the hydrogen bonding between formaldehyde and AM is shown in Fig. 5d. The molar ratio of the functional monomer and crosslinker was defined as w = mol (AM):mol (ALFO sol) = 40:100. First, 1.0 mmol of formaldehyde was mixed with 4.0 mmol of AM, treated with ultrasonication for 30 min, and left for 8 h to form solution B_f_. Then, 1.0 mmol of AIBN was dissolved in 20 mL of formaldehyde and mixed with solution B_f_ and ALFO sol (10.0 mmol). The final mixture was treated with ultrasonication for 30 min, stirred at 50 °C for 12 h under a nitrogen atmosphere under reflux and then dried. Finally, the xerogel was heated at 800 °C for 2 h, yielding Mater.f.

### Fabrication of the sensor array

The prepared materials were further mixed with distilled water and ground to form a paste, which was subsequently printed onto an alumina tube. Two Au electrodes were placed at the ends of the tube. The length of the alumina tube was 4 mm, and the diameter was 1.2 mm. To improve their stability and repeatability, the gas sensors were aged at 150 °C for 170 h in air. The gas-sensing properties were tested using a WS-30A gas senor tester. The relative humidity of the air chest was adjusted by injecting different amounts of water onto a hot plate. As the water was vaporized, the relative humidity changed and was monitored using a hygrometer.

### Characterization

The XRD patterns were obtained for phase identification with a D/max23 diffractometer using Cu Kα1 radiation (λ = 1.54056 Å); the diffracted X-ray intensities were recorded as a function of 2θ. The accelerating voltage was 35 kV, the applied current was 25 mA, and the sample was scanned from 20° to 80° (2θ) in 0.02° steps. The functional groups were identified by FTIR (FTS-40), and each sample was embedded in a KBr pellet and scanned from 4000 cm^−1^ to 400 cm^−1^. The particle morphology of each sample was determined by TEM (JEM-2100). The surface morphology and fracture surface were characterized by scanning electron microscopy (SEM, FEI Quanta 200). The pore size and Brunauer-Emmett-Teller (BET) surface area were analyzed using Quantachrome QuadRASORB-evo equipment.

## Electronic supplementary material

Supporting Information
