# Supplementary material for: A gas sensor array for the simultaneous detection of multiple VOCs
Source: Sci Rep. 2017 May 16;7:1960. doi: 10.1038/s41598-017-02150-z (PMC5434030; doi:10.1038/s41598-017-02150-z)
Supplement: Supplementary file 1 — Supporting Information [file 41598_2017_2150_MOESM1_ESM.doc]

Supporting Information

A gas sensor array for simultaneous detection of multiple VOCs

*Yumin Zhang, Jianhong Zhao, Tengfei Du, Zhongqi Zhu, Jin Zhang* & Qingju Liu**

Supporting Figures


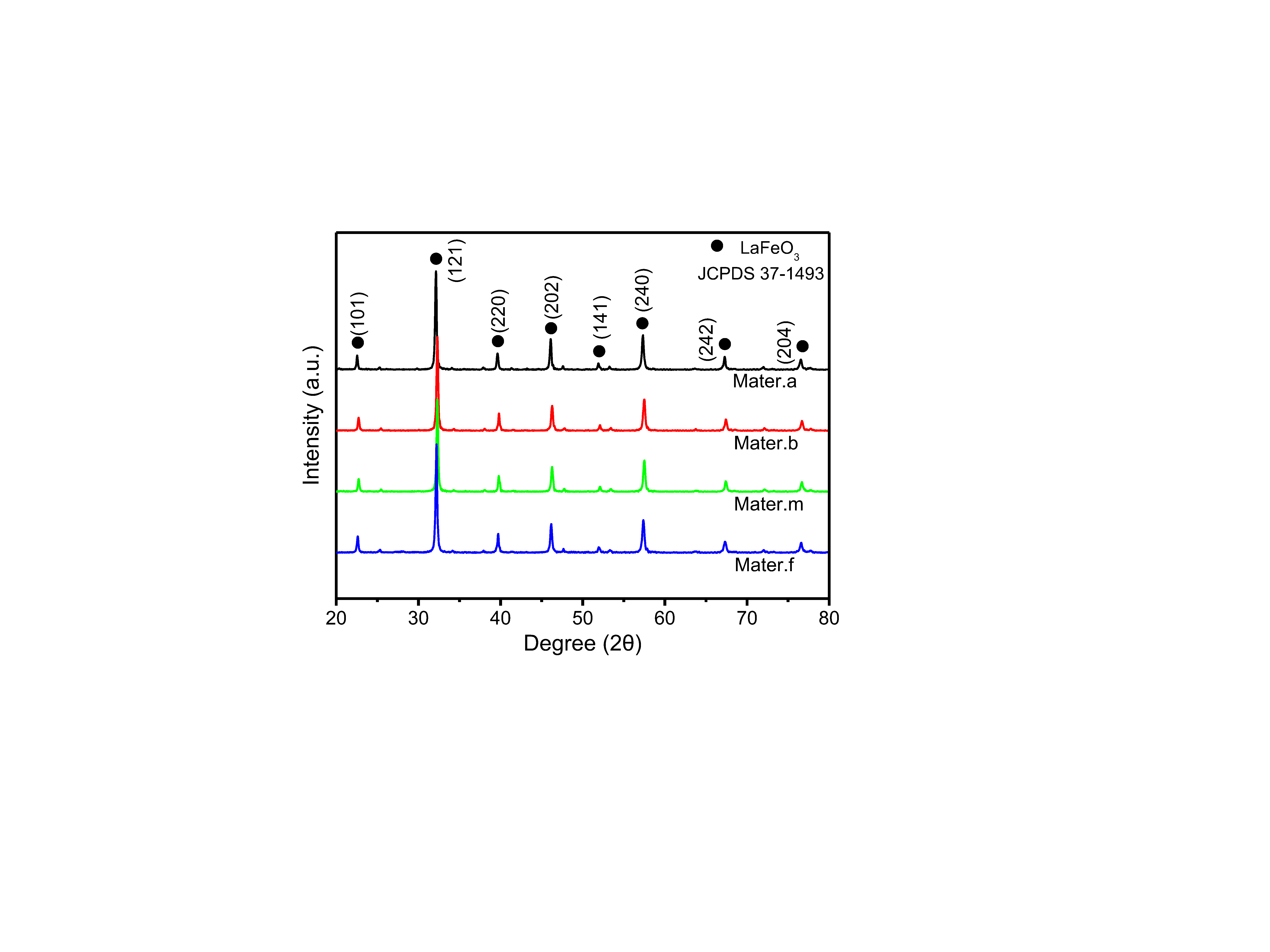


**Figure S1.** XRD patterns of Mater.a, Mater.b, Mater.m and Mater.f.


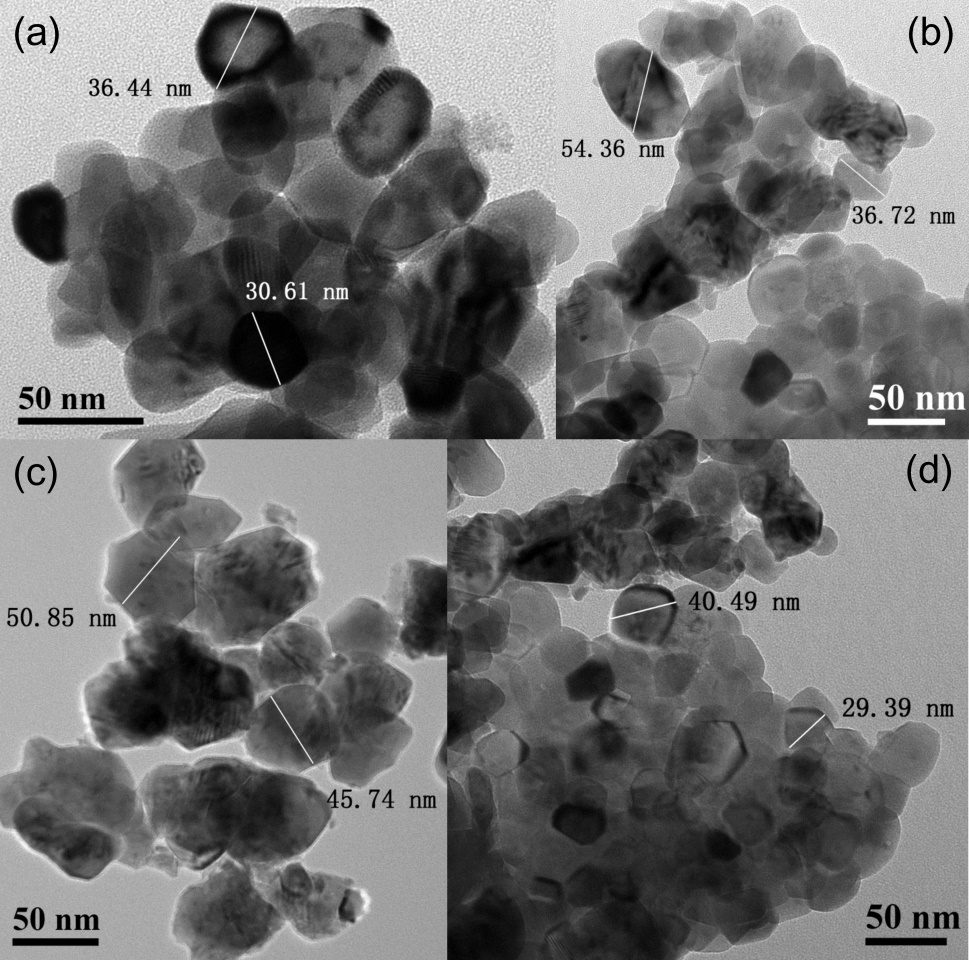


**Figure S2.** TEM images of a) Mater.a, b) Mater.b, c) Mater.m and d) Mater.f.


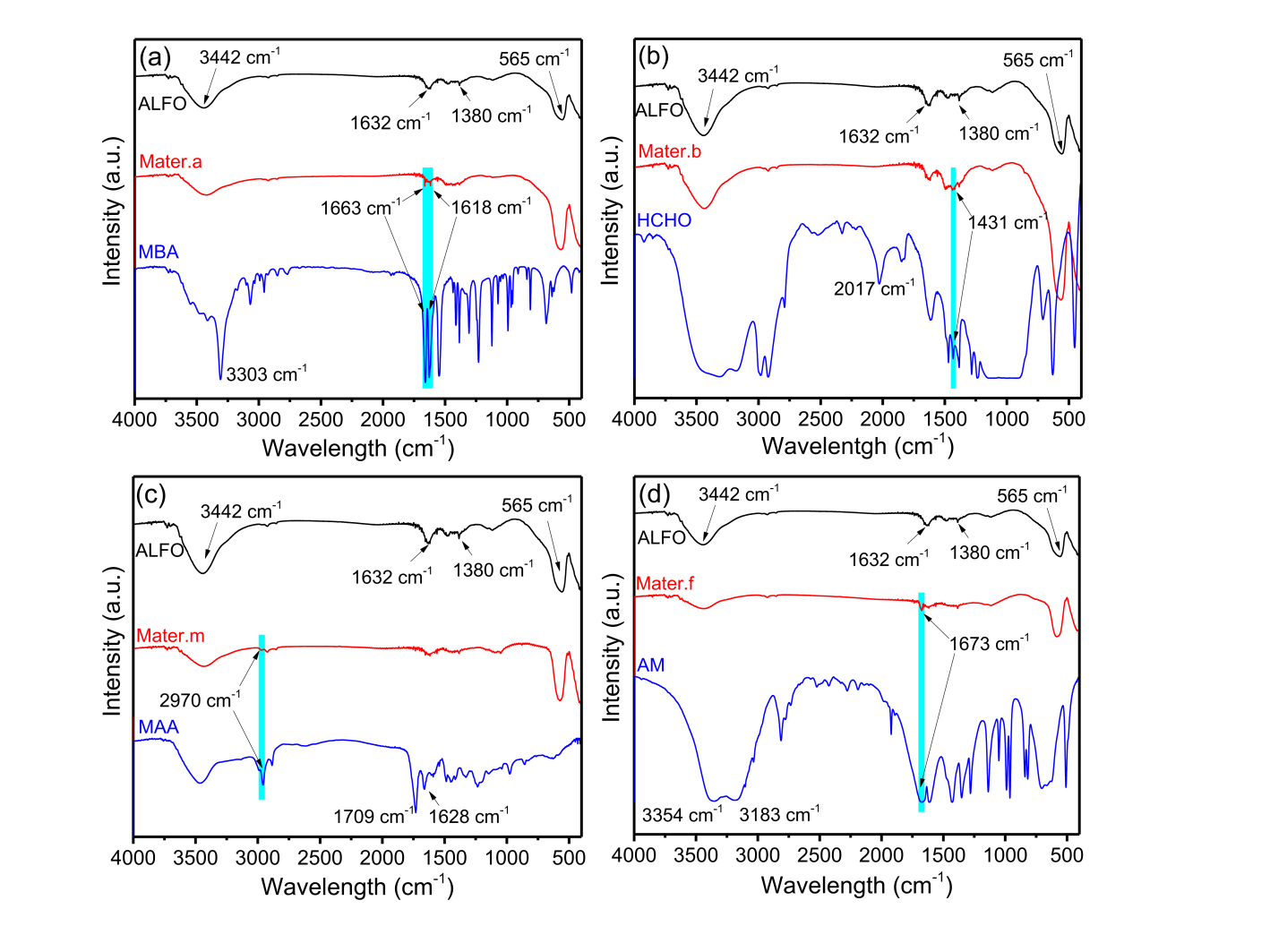


**Figure S3.** Infrared spectra of each material and its related functional monomer, a) infrared spectra of ALFO, Mater.a and MBA, b) infrared spectra of ALFO, Mater.b and HCHO, c) infrared spectra of ALFO, Mater.m and MAA, d) infrared spectra of ALFO, Mater.f and AM.

**Figure S3** illustrates the FT-IR spectroscopy of ALFO, acetone, benzene, methanol and formaldehyde and their related functional monomer N,N'-Methylenebisacrylamide (MBA), formaldehyde (FA), methacrylic acid (MAA) and acrylamide (AM). In the curve of ALFO, the peaks around 565 cm^-1^, 3442 cm^-1^ indicate Fe-O vibrations and the stretching vibration of O-H ^1,2^ of H_2_O in air respectively, and the peaks around 1632 cm^-1^ and 1380 cm^-1^ are attributed to the La-O vibrations^3^. Compared with the curve of ALFO, in the curve of Mater.a (Figure S3a), new peaks at 1663 and 1618 cm^-1^ have appeared, which is attributed to the stretching vibration of C=C in MBA^4,5^. Compared with the curves of Mater.a and MBA, the weakening of the relatively strong peak of C=C stretching vibration (1663 and 1618 cm^-1^) and the disappearance of N-H stretching vibration (3303 cm^-1^ ^5^) in amidogen of Mater.a suggest the successful interaction between ALFO and MBA, and the interaction should be ascribed to the coordination between amidogen groups in MBA and La in ALFO^6^. Similarly, in the curve of Mater.b (Figure S3b), new peak at 1431 cm^-1^ has appeared compared with the curve of ALFO, which is attributed to the bending of C-H in FA^7,8^. Compared the curves of Mater.b and FA, the weakening of the relatively strong peak of C-H bending vibration (1431 cm^-1^) and the disappearance of C=O stretching vibration (2017 cm^-1^) of Mater.b suggest the successful interaction between ALFO and FA, and the interaction should be ascribed to the coordination between carbonyl groups in FA and La in ALFO^6^. In the curve of Mater.m (Figure S3c), new peaks appearing at 2970 cm^-1^ compared with the curve of ALFO, which are attributed to the stretching vibration of O-H in carboxylic acid^9^. Compared the curves of Mater.m and MAA, the weakening of the relatively strong peak of O-H (1210 cm^-1^) stretching vibration and the disappearance of C=O (1631 cm^-1^) stretching vibration in carboxylate anion and C=O (1710 cm^-1^) stretching vibration in carboxylic acid suggests the successful interaction between ALFO and MAA, and the interaction should be ascribed to the coordination between carbonyl groups in MAA and La in ALFO^3,6^. In the curve of Mater.f (Figure S3d), new peak at 1680 cm^-1^ has appeared compared with the curve of ALFO, which is attributed to the stretching vibration of C=O in amidogen^10,11^. Compared the curves of Mater.f and AM, the weakening of the relatively strong peak of C=O stretching vibration (1680 cm^-1^) and the disappearance of N-H stretching vibration (3183 cm^-1^ and 3354 cm^-1 12^) in amidogen of Mater.f suggest the successful interaction between ALFO and AM, and the interaction should be ascribed to the coordination between amidogen groups in AM and La in ALFO^6^.


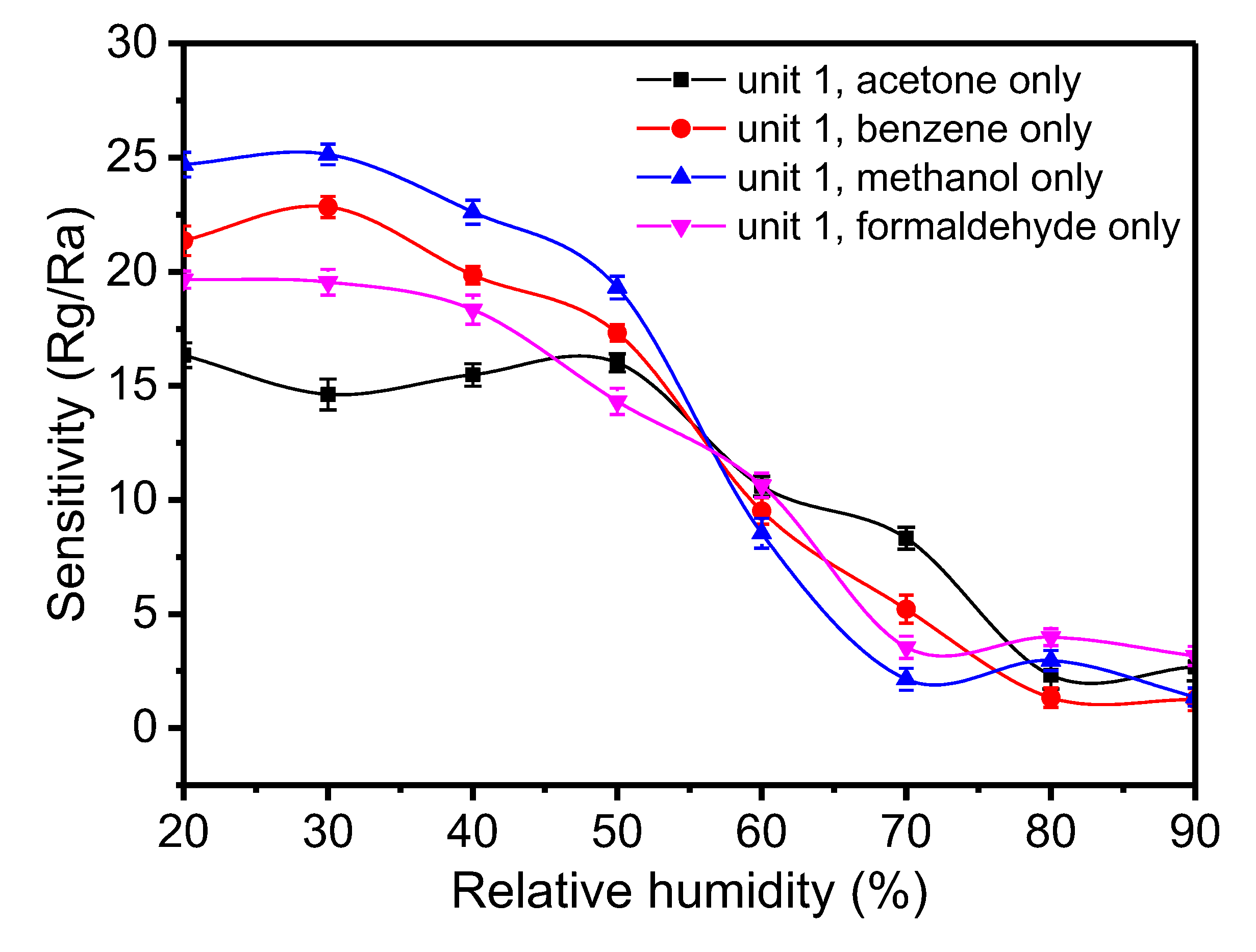


**Figure S4.** Variation trend of the sensitivity of each unit under different relative humidity. The ideal relative humidity that the array could operate normally and reasonably is 20% to 50%. It would be passivated when the relative humidity is higher than 70%.


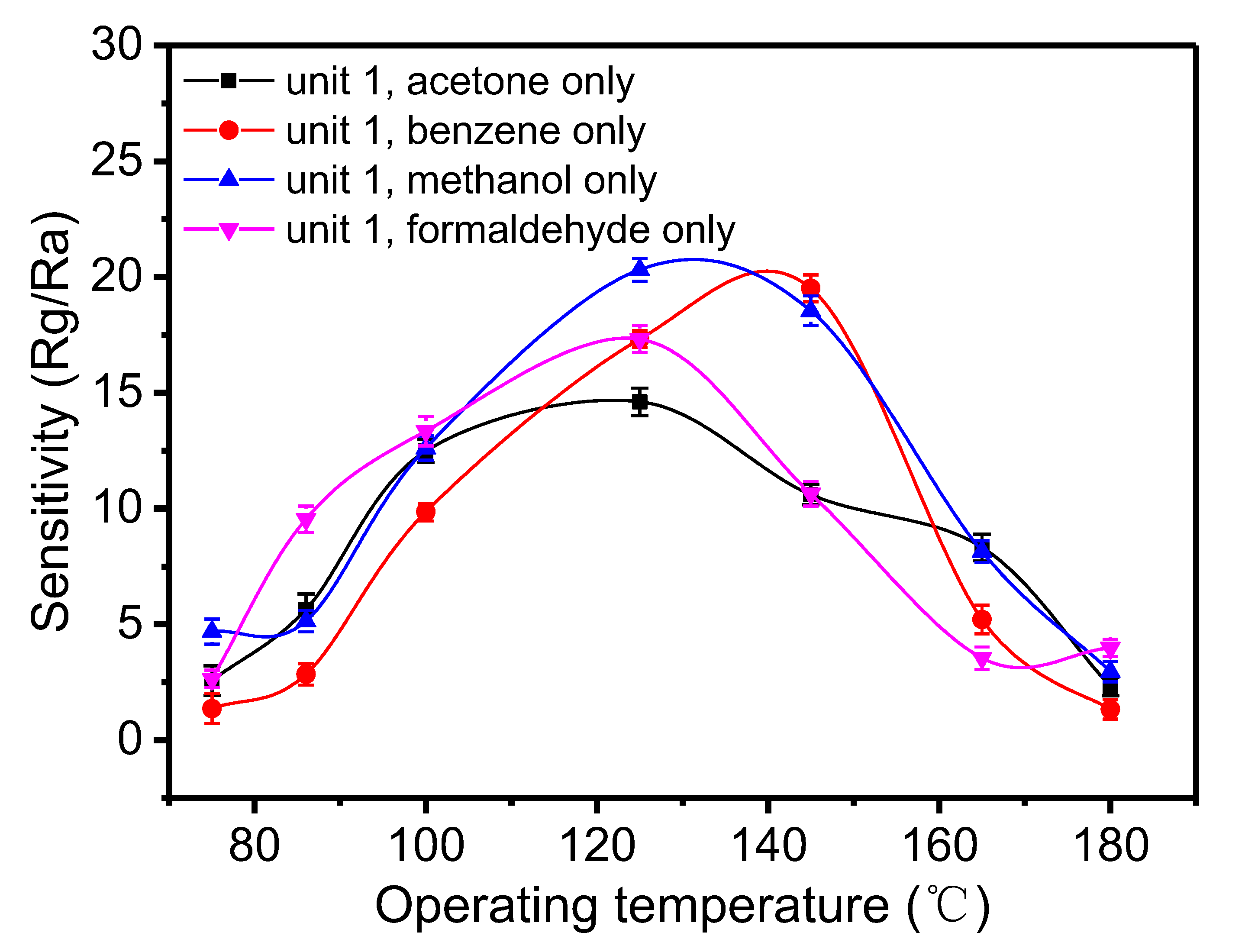


**Figure S5.** Variation trend of the sensitivity of each unit under different operating temperature. The ideal operating temperature that the array could operate normally and reasonably is 125℃.


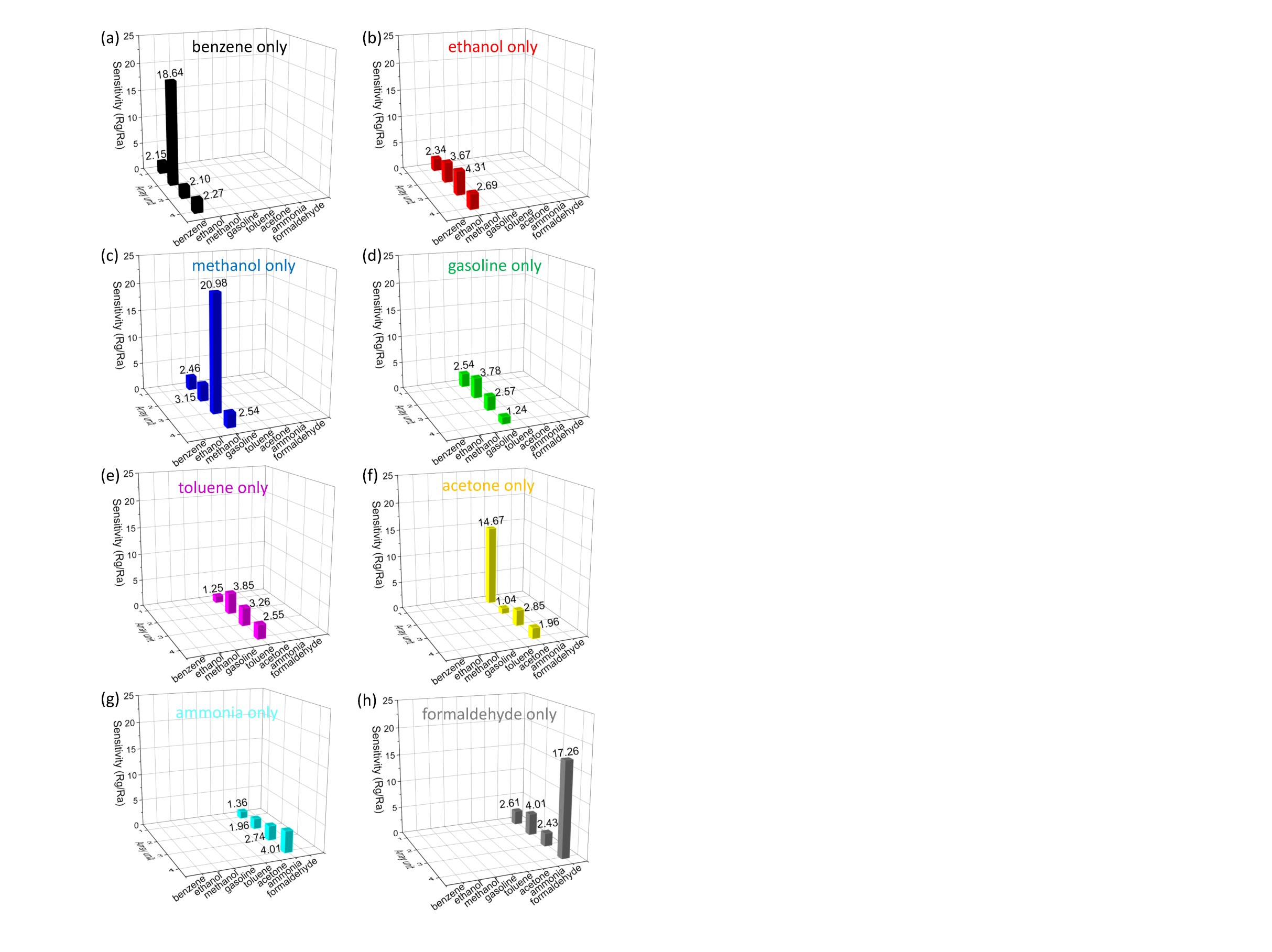


**Figure S6.** Sensitivity of the array to each single analyte. a) to benzene only. Unit 2 exhibit the highest sensitivity to benzene, b) to ethanol only, c) to methanol only. Unit 3 exhibit the highest sensitivity to methanol, d) to gasoline only; e. to toluene only, f) to acetone only. Unit 1 exhibit the highest sensitivity to acetone, g) to ammonia only, h) to formaldehyde only. Unit 4 exhibit the highest sensitivity to formaldehyde.


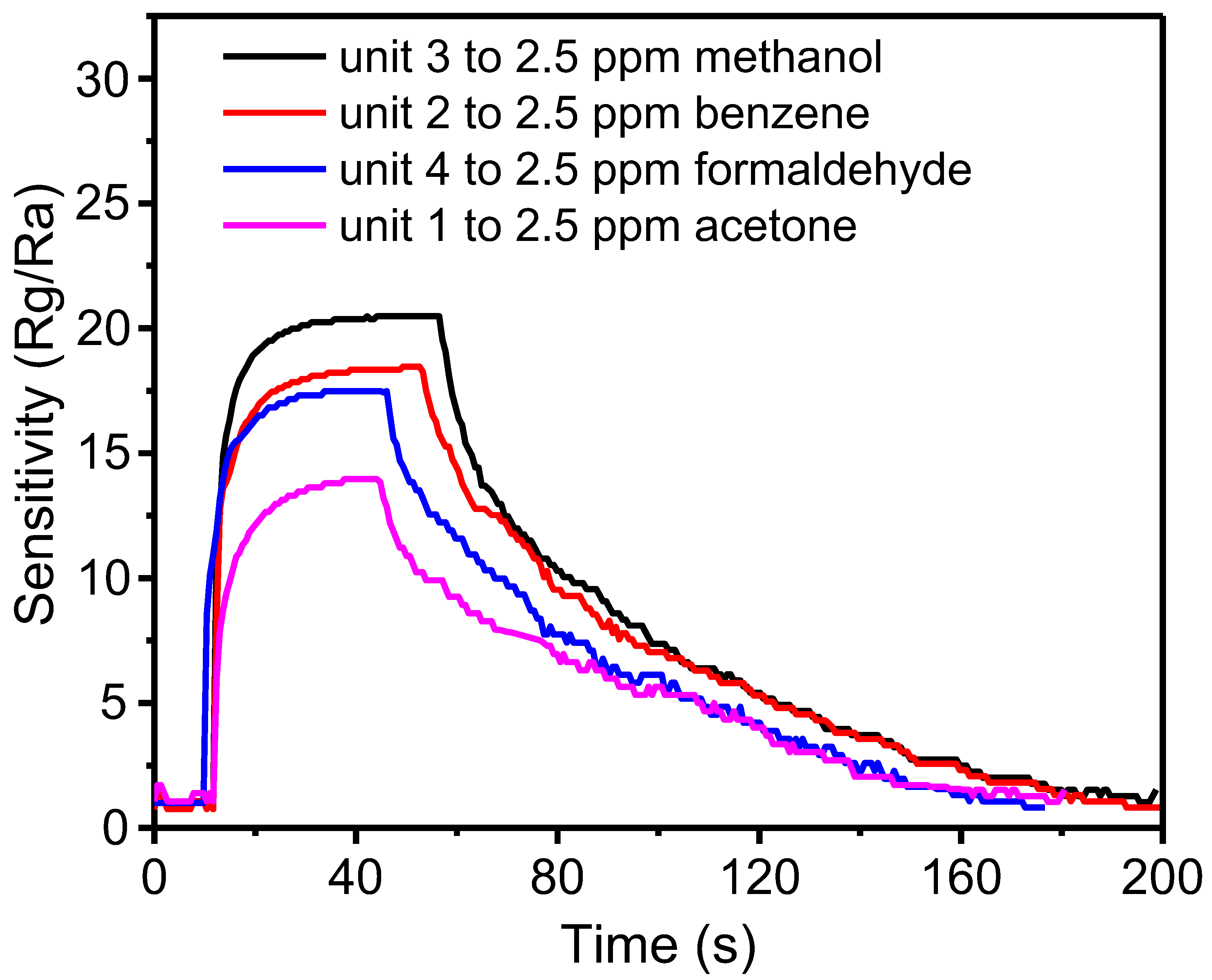


**Figure S7.** The response and recovery time of the array.

**Supporting Tables**

| **Materials** | **Test analyte** | **Concentration**  **(ppm)** | **Response**  **(Rg/Ra or Ra/Rg)** | **Operating**  **temperature (℃)** | **Reference** |
| --- | --- | --- | --- | --- | --- |
| 3D Sn-ZnO | acetone | 100 | 600 | 300 | 13 |
| Flower-like SnO_2_ | acetone | 100 | 40 | 300 | 14 |
| TiO_2_–NiO | acetone | 200 | 10 | 400 | 15 |
| ALFO | acetone | 2.5 | 14.67 | 125 | This work |
| Au-ZnO | benzene/toluene | 10/10 | 3.7/6.3 | 340 | 16 |
| ALFO | benzene | 2.5 | 18.64 | 125 | This work |
| CNTs-SnO_2_ | methanol | 1000 | 1000 | 200 | 17 |
| SnO_2_-Pd-Pt-In_2_O_3_ | methanol | 100 | 320.73 | 160 | 18 |
| honeycomb-like SnO_2_ | methanol | 50 | 7.7 | 320 | 19 |
| ALFO | methanol | 2.5 | 20.98 | 125 | This work |
| SnO_2_ | formaldehyde | 100 | 38.28 | 200 | 20 |
| Cr-WO_3_ | formaldehyde | 50 | 1.8 | 200 | 21 |
| porous In_2_O_3_ | formaldehyde | 50 | 86 | 130 | 22 |
| ALFO | formaldehyde | 2.5 | 17.26 | 125 | This work |

**Table S1**. Summary of previous studies in this field

| **Unit** | **BET area**  **(m^2^ g^-1^)** | **Pore volume**  **(cc g^-1^)** | **Average pore diameter**  **(nm)** |
| --- | --- | --- | --- |
|
| Mater.a (Unit 1) | 6.710 | 0.051 | 38.67 |
| Mater.b (Unit 2) | 4.184 | 0.045 | 39.38 |
| Mater.m (Unit 3) | 6.450 | 0.047 | 44.52 |
| Mater.f (Unit 4) | 3.923 | 0.038 | 34.25 |

**Table S2.** BET area, pore volume and average pore diameter of the four units in the array

References

1. Karlsson M. *et al.* Vibrational properties of proton conducting double perovskites, *Solid State Ionics* **176,** 2971-2974 (2005).

2. Andoulsi-Fezei R. *et al.* Influence of zinc incorporation on the structure and conductivity of lanthanum ferrite, *Ceram. Int.* **42**, 1373-1378 (2016).

3. Cheng X. *et al.* Synthesis and Crystal Structure of Carboxyl Oxygen-Bridged La (III) Four-nuclear Complex [C_48_H_60_La_4_O_35_], *Chem. Bull*. **70**, 861-864 (2007).

4. Chen Q. D. *et al.* Synthesis of poly (N,N '-methylenebisacrylamide-co-4- vinylpyridine) microgels by gamma-ray irradiation, *Acta Polym Sin* 60-65 (2005).

5. Huang X. *et al.* Preparation, characterization and application of a new stir bar sorptive extraction based on poly (vinylphthalimide-co-N, N'-methylenebisacrylamide) monolith, *J. Sep. Sci.* **34**, 3418-3425 (2011).

6. Yang J. *et al.* Synthesis and spectroscopic characterization of complexes of trivalent lanthanide ions Eu(Ш) and Tb(Ш), *Spectrosc. Spectral. Anal.* **22**, 741-744 (2002).

7. Haus R. *et al.* Mobile Fourier-transform infrared spectroscopy monitoring of air pollution, *Appl. Opt*. **33**, 5682-5689 (1994).

8. Routray K. *et al.* Catalysis Science of Methanol Oxidation over Iron Vanadate Catalysts: Nature of the Catalytic Active Sites, *Acs Catalysis* **1**, 54-66 (2011).

9. Zhu Y. *et al.* Surface modification of polycaprolactone with poly(methacrylic acid) and gelatin covalent immobilization for promoting its cytocompatibility, *Biomaterials* **23**, 4889-4895 (2002).

10. Jiang F. *et al.* A double-tailed acrylamide hydrophobically associating polymer: Synthesis, characterization, and solution properties, *J Appl Polym Sci* **132**, 7381–7384 (2015).

11. Martinez-Gomez F. *et al.* Preparation and swelling properties of homopolymeric alginic acid fractions/poly(N-isopropyl acrylamide) graft copolymers, *J. Appl. Polym. Sci*. **132**, (2015).

12. Gunter W. Enzyme-like catalysis by molecularly imprinted polymers, *Chem. Rev.* **102**, 1-27 (2002).

13. Zhang, G. H. *et al.* CTAB-assisted synthesis of 3D Sn doped ZnO nanostructures with enhanced acetone sensing performance, *Mater. Lett.* **162**, 265-268 (2016).

14. Zeng, Y. *et al.* Synthesis and the improved sensing properties of hierarchical SnO_2_ hollow nanosheets with mesoporous and multilayered interiors, *Sens. Actuators B* **222**, 354-361 (2016).

15. Sun, G.-J. *et al.* Synthesis of TiO_2_ nanorods decorated with NiO nanopartieles and their acetone sensing properties, *Ceram. Int*. **42**, 1063-1069 (2016).

16. Wang, *et al.* A Au-functionalized ZnO nanowire gas sensor for detection of benzene and toluene, *Phys. Chem. Chem. Phys.* **15**, 17179-17186 (2013).

17. Aroutiounian, V. M. *et al.* Study of the surface-ruthenated SnO_2_/MWCNTs nanocomposite thick-film gas sensors, *Sens. Actuators B* **177**, 308-315 (2013).

18. Li, Y. *et al.* A high performance methanol gas sensor based on palladium-platinum-In_2_O_3_ composited nanocrystalline SnO_2_, *Sens. Actuators B* **237**, 133-141 (2016).

19. Wang, L. L. *et al.* Methanol sensing properties of honeycomb-like SnO_2_ grown on silicon nanoporous pillar array, *J. Alloy. Compd.* **682**, 170-175 (2016).

20. Li, Y. *et al.* Formaldehyde detection: SnO_2_ microspheres for formaldehyde gas sensor with high sensitivity, fast response/recovery and good selectivity, *Sens. Actuators B* **238**, 264-273 (2017).

21. Upadhyay, S. B., Mishra, R. K. & Sahay, P. P. Cr-doped WO_3_ nanosheets: Structural, optical and formaldehyde sensing properties, *Ceram. Int.* **42**, 15301-15310 (2016).

22. Wang, J. *et al.* Microstructure and gas sensing property of porous spherical In_2_O_3_ particles prepared by hydrothermal method, *Powder Technol.* **303**, 138-146 (2016).
